# Rapid scale-up of COVID-19 training for frontline health workers in 11 African countries

**DOI:** 10.1186/s12960-022-00739-8

**Published:** 2022-05-16

**Authors:** Fatima Tsiouris, Kieran Hartsough, Michelle Poimboeuf, Claire Raether, Mansoor Farahani, Thais Ferreira, Collins Kamanzi, Joana Maria, Majoric Nshimirimana, Job Mwanza, Amon Njenga, Doris Odera, Lyson Tenthani, Onyekachi Ukaejiofo, Debrah Vambe, Erika Fazito, Leena Patel, Christopher Lee, Susan Michaels-Strasser, Miriam Rabkin

**Affiliations:** 1 ICAP at Columbia University, New York, NY United States of America; 2 Resolve to Save Lives, An Initiative of Vital Strategies, New York, NY United States of America; 3 Mailman School of Public Health, Columbia University, New York, NY United States of America; 4 HRH Training Unit, Mailman School of Public Health, ICAP at Columbia University, 722 West 168th Street, New York, NY 10032 United States of America

**Keywords:** COVID-19 training, Curriculum development, Virtual training, HRH Development, Competency-based training, Africa, Health workforce, IPC

## Abstract

**Background:**

The global spread of the SARS-CoV-2 virus highlights both the importance of frontline healthcare workers (HCW) in pandemic response and their heightened vulnerability during infectious disease outbreaks. Adequate preparation, including the development of human resources for health (HRH) is essential to an effective response. ICAP at Columbia University (ICAP) partnered with Resolve to Save Lives and MOHs to design an emergency training initiative for frontline HCW in 11 African countries, using a competency-based backward-design approach and tailoring training delivery and health facility selection based on country context, location and known COVID-19 community transmission.

**Methods:**

Pre- and post-test assessments were conducted on participants completing the COVID-19 training. Parametric and non-parametric methods were used to examine average individual-level changes from pre- to post-test, and compare performance between countries, cadres, sex and facility types. A post-evaluation online training survey using Qualtrics was distributed to assess participants’ satisfaction and explore training relevance and impact on their ability to address COVID-19 in their facilities and communities.

**Results:**

A total of 8797 HCW at 945 health facilities were trained between June 2020 and October 2020. Training duration ranged from 1 to 8 days (median: 3 days) and consisted of in person, virtual or self guided training. Of the 8105 (92%) HCW working at health facilities, the majority (62%) worked at secondary level facilities as these were the HF targeted for COVID-19 patients. Paired pre- and post-test results were available for 2370 (25%) trainees, and 1768 (18%) participants completed the post-evaluation training survey. On average, participants increased their pre- to post-test scores by 15 percentage points (95% CI 0.14, 0.15). While confidence in their ability to manage COVID-19 was high following the training, respondents reported that lack of access to testing kits (55%) and PPE (50%), limited space in the facility to isolate patients (45%), and understaffing (39%) were major barriers.

**Conclusion:**

Ongoing investment in health systems and focused attention to health workforce capacity building is critical to outbreak response. Successful implementation of an emergency response training such as this short-term IPC training initiative in response to the COVID-19 pandemic, requires speed, rigor and flexibility of its design and delivery while building on pre-existing systems, resources, and partnerships.

## Background

The global spread of the SARS-CoV-2 virus highlights both the importance of frontline healthcare workers (HCW) in pandemic response and their heightened vulnerability during infectious disease outbreaks. Health systems require competent, well-equipped and widely distributed HCW to diagnose and treat new diseases, prevent transmission within health facilities, and communicate accurate health messaging to the populations they serve [1]. Yet without up-to-date knowledge and the resources and skills required for effective infection prevention and control (IPC), HCW battling novel pathogens are at increased risk of exposure and death [2, 3].

The longstanding global consensus on the need to train, retain and support more frontline HCW has been reinforced by the stark warnings of recent outbreaks in which HCW died in disproportionate numbers compared to the general population, including SARS (2002–2003), MERS (2012) and Ebola virus disease (2014–2016) [4, 5]. The West African Ebola outbreak underscored the point that HCW are vulnerable to morbidity and mortality, especially in the early response phase [6]. This pattern is repeating itself during the COVID-19 pandemic; despite limited data, it is clear that hundreds of thousands of frontline HCW have contracted COVID-19 and tens of thousands have died [7–11]. Many frontline HCW remain at risk due to lack of proper personal protective equipment (PPE).

Optimizing epidemic response requires ongoing investment in health systems to enable surveillance for new pathogens, adequate water and sanitation infrastructure, access to appropriate PPE and supplies, and the pre-service education, in-service training and ongoing supportive supervision needed to ensure HCW knowledge and skills. In addition to ensuring that HCW are competent to prevent, detect and respond to known health threats, health systems need a way to swiftly equip frontline HCW with information about novel and emergent pathogens and to develop competence amidst crises. This can be particularly challenging in resource-limited settings, where HCW are scarce and often maldistributed, and in-service training and opportunities for professional development are limited [12–14].

Adequate preparation, including the development of human resources for health is essential to an effective response. Many lessons about HCW training and capacity building were learned from the 2014–2016 Ebola epidemic, including the lack of adequate IPC training for frontline HCW, the need to quickly reorient HCW to new modes of working, the importance of rapid refresher trainings as information about emerging pathogens evolves with experience, and the need for HCW training on strategies to maintain essential health services and provide effective community engagement [15]. In the years following the West Africa Ebola outbreak, the global community committed to substantial investments in global health security and in expanding, strengthening and empowering the health workforce in resource-limited settings [16, 17]. For example, across Africa, continuing professional development is increasingly required as part of re-licensure [14]. However, despite advances in the development of emergency response strategies, surveillance and warning systems and national IPC policies, guidelines and training curricula, the emergence of the COVID-19 pandemic in 2020 made it clear that significant gaps remained [18, 19].

When the first case of COVID-19 was reported in Africa in February 2020, millions of HCW across the continent needed to learn about the new disease and to improve their basic IPC knowledge and skills. While the 2019 Global Health Security Index found that no country was fully prepared for a pandemic, the African region had the lowest scores [20]. Recent studies from South Africa [19], Nigeria [21], Ethiopia [22] and Libya [23] illustrate the need for improved coverage of basic IPC training among frontline HCW of all cadres, an initiative which should ideally be built into both pre-service education and in-service training in all countries. Training alone is insufficient to close the IPC performance gap—enabling policies, guidelines, systems, supplies, and infrastructure are also essential [24]. But training, supportive supervision, clear communication, and supportive workplace culture are critically important enablers of HCW IPC performance [25].

In addition to sustained and integrated education and training on epidemic preparedness and response, HCW need access to rapid knowledge transfer during outbreaks and emergencies. In the early months of the COVID response, many organizations hosted training webinars and videos, including the World Health Organization (WHO), the U.S. Centers for Disease Control and Prevention (CDC), Africa CDC, the African Society of Laboratory Medicine (ASLM), the Africa One Health University Network (AFROHUN), the Infection Control Africa Network (ICAN) and others. But Ministries of Health (MOHs) across the continent lacked easy access to COVID-19-specific training courses for frontline HCW at primary and secondary health facilities or the ability to rapidly deliver training at scale to these vital cadres.

In response [26], ICAP at Columbia University (ICAP) partnered with Resolve to Save Lives and MOHs to design an emergency training initiative for frontline HCW in 11 African countries, using a competency-based backward-design approach and tailoring training delivery to country context. Adopting a backward design emphasized the need to clearly articulate learning goals beyond what health workers need to *know* to what they need to *know how to do* [27]. The curriculum focused on practical knowledge and skills and included modules on the identification and implementation of IPC strategies, patient screening and triage, maintenance of essential services during a pandemic, and community outreach and communication. From June 2020 through October 2020, the curriculum was used to train 8797 frontline HCW in Angola, Burundi, Eswatini, Kenya, Lesotho, Malawi, Mozambique, Rwanda, Sierra Leone, South Sudan, and Zambia.

## Methods

### Training design and implementation

Following consultation with MOHs and other local stakeholders, we conducted a review of existing training materials, global and national guidelines, and country-specific resources focused on COVID-19 training. After this review, we developed a competency framework outlining the basic knowledge and skills that frontline HCW in low-resource settings need to confront the COVID-19 pandemic (Table 1). Using the backward design approach to curriculum development, we then identified functional competencies and how each competency should ideally be taught and assessed. The result was a standardized training package consisting of 10 training modules, each with PowerPoint slides and speakers’ notes, assessment tools including a pre/post-test, and a resource library of up-to-date guidance, job aides, and supplemental training materials from other organizations (e.g., WHO, Africa CDC). Training methods included a mixture of didactic lectures and case studies, brief knowledge-check questions, reflection questions, and, where feasible, simulation of key skills such as donning and doffing PPE.

**Table 1:** COVID-19 training package

Domain	Competency
1. COVID-19 Origins	HCW can recognize key characteristics of the novel coronavirus and can describe their country’s COVID-19 epidemic stage
2. Disease transmission	HCW can recognize the signs and symptoms of COVID-19 and can list the criteria for suspected, probable, and confirmed cases. They can test suspected cases following national guidelines and report confirmed cases using national reporting tools and platforms
3a. Infection prevention and control (IPC) overview	HCW can ensure adequate IPC including prevention of infections among HCW and nosocomial transmission of COVID-19 within health facilities. Healthcare workers understand IPC principles and practices and how they are applied to the COVID-19 situation
3b. Standard and transmission precautions	HCW can implement standard and transmission-based precautions as per facility standards and guidelines
3c. Implementation strategies	HCW can describe and implement environmental controls to minimize the spread of COVID-19 within health facilities
3d. IPC and WASH (water, sanitation and hygiene)	HCW can describe and support administrative controls to minimize the spread of COVID-19 within health facilities
3e. Personal protective equipment (PPE)	HCW can describe and implement correct use of personal protective equipment (PPE) to minimize the spread of COVID-19 within health facilities
4. Triage of COVID-19 patients	HCW can apply knowledge of national screening guidelines to conduct effective triage, including risk stratification, isolation, and patient referral. (From https://preventepidemics.org/wp-content/uploads/2020/07/CCC_022_Tools-for-Primary-Health-Centers_040720-1.pdf)
5. Maintenance of essential services	HCW can understand the impact of COVID-19 and the COVID-19 response on essential health services and can support new health facility protocols to maintain services during the pandemic
6. Effective communication: dispelling myths	HCW can effectively communicate with patients, community member, facility managers and other stakeholders to disseminate key messages on COVID-19 disease including signs and symptoms, when to seek care at a health facility as well as respiratory and hand hygiene

**Fig. 1:**
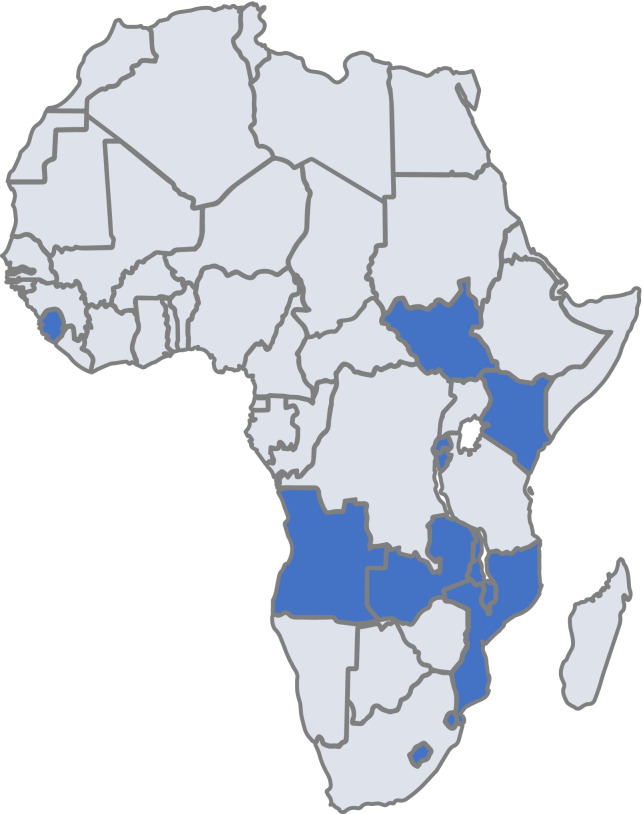
Country training locations

Following development of the “generic” modules in May of 2020, we worked with MOHs and local stakeholders to adapt the training to each of the 11 country contexts. All countries used the competency framework to guide their training curriculum, but there was variability in the content covered based on country priorities and target audience (e.g., targeting community health workers in addition to facility-based health staff). Countries also adapted the delivery modality, language, and training strategy to fit local context. Examples of delivery strategies included in-person training adhering to appropriate safety protocols (social distancing and mask-wearing); live (synchronous) distance training using the Zoom™ platform; asynchronous training that participants completed at their own pace using voice over PowerPoint or the Articulate™ online training platform, or a hybrid of these approaches (Fig. 1).

### Setting and participant selection

All countries targeted frontline HCW, mainly clinical staff and public health practitioners working in health facilities where COVID-19 cases were expected. Facilities were selected in collaboration with local MOHs and other key local stakeholders and were based on location (proximity to borders), catchment population and/or known COVID-19 community transmission. Trainees were selected by health facility managers, and included physicians, nurses, other health facility-based staff, community health workers, health managers and others.

### Data collection, management and analysis

Data were collected from three primary sources; training registration forms, a pre/post-test, and a post-training online survey. Trainee demographic data, including gender, cadre, health facility and country were collected on sign-in sheets for in-person trainings and via virtual registration forms for online trainings. ICAP staff then entered aggregate training data into a customized DHIS2 database that was reviewed for completeness and accuracy by data specialists and project leads in each country. Descriptive analyses of training data were conducted within DHIS2 and Excel.

Training evaluations to assess training satisfaction, knowledge and skills as well as self efficacy were conducted. Eight of the 11 countries included both pre-tests (administered prior to the training) and post-tests (administered immediately following the training) as part of their training program; of these, Kenya utilized pre-tests and post-tests before and after each module. South Sudan and Mozambique opted not to conduct knowledge assessments and Eswatini only conducted a post-test. Countries adapted the pre- and post-test template, so the instruments used were not identical, ranging from 24 questions in Burundi to 40 questions in Sierra Leone and administered online in Kenya and on paper in the other 7 countries. Pre and post-test results were entered into Excel by country-based staff and basic descriptive statistics were created. Only individuals with matched pre- and post-assessment data were included in subsequent analyses. For the purpose of some analyses, a passing score of 70% was established post hoc, since not all countries had established a passing/failing grade. Parametric and non-parametric methods were used to examine average individual-level changes from pre- to post-test, and to compare performance between countries, cadres, sex and facility types. Paired *t*-tests were used to evaluate statistically significant changes in test scores (Table 3). Non-parametric methods were used to evaluate group-level differences in performance and included: Mann–Whitney *U* tests to compare nurses to doctors, men to women, and primary to secondary facilities; and a Kruskal–Wallis test to compare gain scores across all cadres. Significance of *P* < 0.05 was used for the statistical methods.

In September 2020, an online post-training survey was shared with all trainees who had provided an email address and/or contact information when registering for the training course. The 25-question survey was created in Qualtrics™ and was translated into French and Portuguese. It included a mix of closed-ended and open-ended questions designed to assess participants’ satisfaction with the training and to explore its relevance and impact on their ability to address COVID-19 in their facilities and communities. Trainees received a link to the survey via email and/or WhatsApp as well as follow-up reminders. Results from the open-ended questions were reviewed by two researchers for common themes, which were used to develop codes to categorize responses, and the open-ended results were then coded. Descriptive and bivariate analyses were conducted. All analyses were performed with SAS software (version 9.4).

### Ethical review

The project received non-research determination from the Columbia University Institutional Review Board and ethical approvals from all eleven countries. All data were deidentified. As a project with non-research determination reporting only aggregate anonymized data, no individual consent was obtained, and participation was voluntary.

## Results

### Training implementation

Training took place in 11 countries reaching 8797 HCW at 945 health facilities between June 2020 and October 2020. Training duration ranged from 1 to 8 days, with a median length of 3 days. Six countries relied mostly on in-person trainings while adhering to social distancing protocols and country safety guidelines whereas five countries (Eswatini, Kenya, Lesotho, Mozambique, and South Sudan) delivered trainings using a combination of in person and virtual training.

### Participant demographics

Trainees’ demographic and survey completion data are described in Table 2. Most trainees (56%) were nurses and 57% were female. Of the 8105 (92%) working at health facilities, 38% worked at primary level and 62% worked at secondary level. Those not affiliated with health facilities included staff at district or regional health offices, community health care workers, and staff from non-governmental organizations, and ministries of health (*n* = 661). Paired pre- and post-test results were available for 2370 (25%) trainees, and 1768 (18%) participants completed the post-evaluation training survey.

**Table 2:** Overview of training, assessment and survey data demographic characteristics

Demographics	Training *n* = 8797	Pre/post-assessments *n* = 2370	Post-training survey *n* = 1768
Sex *n* (%^a^)			
Female	5017 (57)	1307 (55)	851 (48)
Male	3778 (43)	603 (25)	915 (52)
Other	0 (0)	0 (0)	2 (< 1)
Missing	2 (< 1)	460 (19)	0 (0)
Cadre *n* (%)			
Doctor	712 (8)	229 (10)	126 (7)
Non-clinical	591 (7)	134 (6)	133 (8)
Nurse	4950 (56)	857 (36)	969 (55)
Other HCW	2041 (23)	399 (17)	485 (27)
Community HCW	247 (3)	0 (0)	0 (0)
Other (IP^b^, MOH)	250 (3)	0 (0)	55 (3)
Missing	3 (< 1)	751 (32)	0 (0)
Facility *n* (%)			
Primary	3110 (35)	1314 (55)	698 (40)
Secondary	4995 (57)	653 (28)	691 (39)
Missing	31 (< 1)	403 (17)	0 (0)
N/A/other	661 (8)	0 (0)	379 (21)
Country *n* (%)			
Angola	470 (5)	436 (18)	15 (1)
Burundi	398 (4)	74 (3)	229 (13)
Eswatini	1812 (19)	0 (0)	127 (7)
Kenya	1261 (13)	350 (15)	302 (17)
Lesotho	799 (8)	372 (16)	115 (6)
Malawi	717 (7)	339 (14)	314 (18)
Mozambique	1291 (13)	0 (0)	79 (4)
Rwanda	418 (4)	409 (17)	287 (16)
Sierra Leone	348 (4)	306 (13)	4 (< 1)
South Sudan	804 (8)	0 (0)	200 (11)
Zambia	479 (5)	84 (4)	92 (5)
Missing	0 (0)	0 (0)	4 (< 1)

^a^Percent listed represents column percent

^b^IP = other implementing partners

### Pre/post-test results

Table 3 presents the average pre- and post-test scores by country for the 2370 participants with available paired pre- and post-test data. All individual-level increases from pre to post-test were found to be statistically significant (*p*-value < 0.0001), and the proportion of individuals passing the assessment at a score of 70% increased in all countries. On average, participants increased their score from pre- to post-test by 15 percentage points (95% CI 0.14, 0.15). Participants from Burundi made up the smallest country cohort (*n* = 74, 3.1%), yet had the greatest improvement from pre- to post-test, with an average increase of 42 percentage points from pre- to post-test (95% CI 0.39, 0.45). Participants from Kenya made up the second largest country cohort (*n* = 350, 15%) and scored higher on both the pre- (average pretest score = 80%) and post-test (average post-test score = 89%) than participants from any other country (Table 3).

**Table 3:** Comparison of participant scores in the pre- and post-test and proportions with a passing grade at the 70% cutoff by country

Country	*N*	Mean ± SD			95% CI	*t* value*	Pre-test pass ≥ 70% *N* (%)	Post-test pass ≥ 70% *N* (%)
		Pre-test	Post-test	Increase				
Angola	436	0.57 ± 0.10	0.68 ± 0.10	0.11 ± 0.09	(0.10, 0.12)	25.02	56 (13)	212 (49)
Burundi	74	0.27 ± 0.11	0.69 ± 0.10	0.42 ± 0.12	(0.39, 0.45)	29.76	0 (0)	34 (46)
Kenya	350	0.72 ± 0.14	0.89 ± 0.07	0.17 ± 0.15	(0.16, 0.19)	22.22	203 (58)	350 (100)
Lesotho	372	0.59 ± 0.14	0.69 ± 0.12	0.10 ± 0.10	(0.09, 0.11)	18.85	99 (27)	199 (53)
Malawi	339	0.56 ± 0.11	0.74 ± 0.11	0.18 ± 0.12	(0.17, 0.19)	27.87	35 (10)	235 (69)
Rwanda	409	0.59 ± 0.11	0.73 ± 0.10	0.13 ± 0.12	(0.12, 0.15)	22.97	54 (10)	230 (56)
Sierra Leone	306	0.54 ± 0.14	0.70 ± 0.13	0.16 ± 0.12	(0.15, 0.18)	23.88	31 (10)	176 (58)
Zambia	84	0.62 ± 0.12	0.70 ± 0.10	0.09 ± 0.13	(0.06, 0.11)	6.03	27 (32)	45 (54)
Total	2370	0.59 ± 0.15	0.73 ± 0.13	0.15 ± 0.13	(0.14, 0.13)	55.09	505 (21)	1481 (62)

Results are expressed as mean and standard deviation of scores obtained in pre- and post-tests. Significance obtained using paired *t*-test

*SD* standard deviation

*All paired *t*-tests were highly statistically significant at a *p*-value of < 0.0001Kenya’s pre- and post-tests were distributed by module, not as a singular cumulative test post-exposure to all modules

Performance across participant demographic characteristics varied by cadre, facility type, and sex. As noted in Table 4, the largest cohorts were nurses (*n* = 857) and females (*n* = 1307). Overall, doctors had the highest pass-rate in the post-test (*n* = 193), with 84% of all doctors passing at or above 70%. Nurses had the second highest pass-rate in the post-test (*n* = 559), with 65% of all nurses passing at or above 70%. Nurses also demonstrated a large average improvement from pre to post-test (19 percentage points). There were no statistically significant differences in gain scores comparing HCW from primary (*N* = 1314) to secondary (*N* = 653) facilities (Pr > *Z* 0.1988), females (*N* = 1307) to males (*N* = 603) (Pr > *Z* 0.1524), or nurses (*N* = 857) to doctors (*N* = 229) (Pr > *Z* 0.116); however, there were statistically significant differences in gain scores comparing all cadres using the Kruskal–Wallis Test (*p* = 0.008, 3 DF).

**Table 4:** Comparison of participant pre- and post-test pass/fail proportions at the 70% cutoff by cadre and sex

Characteristic	*N*^a^	Mean ± SD		Pre-test pass ≥ 70% *N* (%)	Post-test pass ≥ 70% *N* (%)
		Pre-test	Post-test		
Cadre^b^					
Doctor	229	0.64 ± 0.14	0.81 ± 0.11	90 (39)	193 (84)
Non-clinical HCW	134	0.51 ± 0.14	0.64 ± 0.14	11 (8)	44 (33)
Nurse	857	0.72 ± 0.14	0.69 ± 0.10	204 (24)	559 (65)
Other HCW	399	0.59 ± 0.14	0.69 ± 0.12	101 (25)	256 (64)
Sex^c^					
Female	1307	0.58 ± 0.15	0.73 ± 0.13	277 (21)	823 (63)
Male	603	0.59 ± 0.16	0.75 ± 0.14	159 (26)	400 (66)

Results are expressed as mean and standard deviation of scores obtained in pre- and post-tests

^a^Represents *N* for those with paired pre- and post-test and demographic data for selected characteristics. Percent listed represents row percent

^b^751 missing cadre information

^c^460 missing sex information

### Post-training survey results

Overall, 1768 participants across the 11 countries responded to the post-training survey (Table 2), the largest proportions of which were from Malawi (18%) and Kenya (17%). The majority of respondents were nurses (55%). Thirteen percent of respondents had less than 1 year of professional experience, 40% had 1–4 years, 24% had 5–10 years, and 23% had more than 10 years. The majority of respondents attended an in-person training (60%), while 14% attended via live webinar, 13% via a combination of methods, 7% through self-study (distance learning), and 6% not specified.

When asked about their satisfaction with the training, of 1560 respondents who answered the question, 1344 (87%) were extremely satisfied or satisfied with the training, while 139 (9%) were neutral, and 77 (5%) were dissatisfied or extremely dissatisfied. For assessment of training duration, among 1560 respondents, 692 (44%) felt that the training was an appropriate amount of time, while 71 (5%) felt it was too long, and 797 (51%) felt that the training was too short. When asked about relevance of training to their daily responsibilities around COVID-19, out of 1569 respondents who answered the question, 1411 (90%) found that the training was extremely relevant or relevant, while 83 (5%) were neutral, and 63 (7%) thought it was not relevant or was extremely irrelevant.

**Fig. 2:**
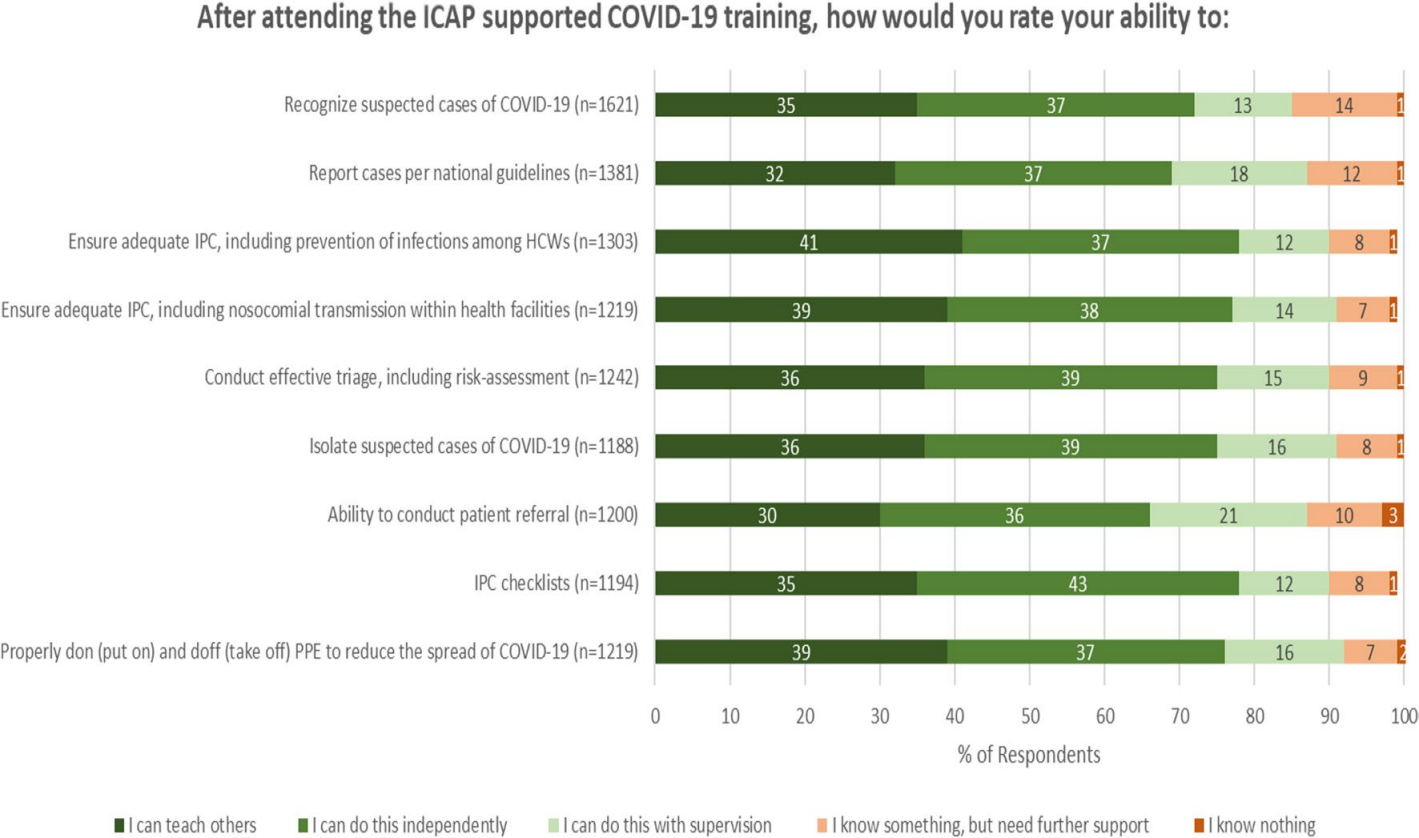
Respondent ratings of abilities post-training

In rating their ability to perform specific IPC-related activities following the training (Fig. 2), the majority of participants indicated that they could either teach others or do the activity independently, with the highest ability ratings in use of IPC checklists, ensuring adequate IPC, including prevention of infections among health care workers, and preventing nosocomial transmission within health facilities (78%, 78%, and 77%, respectively, could teach others or do the activity independently).

Overall respondent confidence in their ability to respond to COVID-19 was high following the training. Among 1572 respondents, 86% were confident or extremely confident in their ability to communicate with community leaders about COVID-19 and to rapidly respond to rumors and myths. Of 1309 respondents, 84% were confident or extremely confident in their ability to communicate with health facility managers about COVID-19 and to rapidly respond to rumors and myths. Among 1271 respondents, 85% were confident or extremely confident in their ability to address COVID-19 at their health facility using the skills and knowledge gained from the ICAP-supported training. And of 1226 respondents, 83% were confident or extremely confident in their ability to continue providing non COVID-19 specific care at their health facility using the skills and knowledge gained from the ICAP-supported training.

Respondents reported that the main barriers limiting their ability to respond to COVID-19 at their health facility included lack of access to testing kits for COVID-19 (55%), lack of access to PPE (50%), limited space in the facility to isolate patients (45%), lack of access to technical equipment (43%), funding or budget constraints (42%), understaffing (39%), and lack of motivation from staff (38%).

Respondents felt that the topics most applicable to their contexts included IPC training on standard and droplet precautions (76%), recognizing COVID-19 signs and symptoms (72%), donning and doffing PPE (61%), and effective communication and community guidance for COVID-19 (59%).

Responses from open-ended questions showed that participants wanted additional training on COVID-19 including refresher courses and trainings on relevant updates around the disease, additional trainings on case management and clinical care, and access to the training materials for future reference. Other themes identified in the open-ended questions included the need for simulation-based training, especially around donning and doffing PPE, as well as the need to expand the training to non-clinical facility staff.

## Discussion

This study describes the development and implementation of a frontline health worker training program in response to the rapid global spread of the novel coronavirus (SARS-CoV-2). The curriculum was swiftly designed in April–May of 2020 and training was delivered to 8797 HCW in 11 countries in 5 months. To respond safely and effectively to the pandemic, modules were developed using a competency-based framework in alignment with WHO evidence-based guidelines for facility and district level IPC training programs. To quickly reach diverse populations across a variety of resource-limited settings, the content and delivery methods were designed to be flexible and adaptable. Training focused on what health workers needed to know and be able to do to respond safely and effectively to COVID-19 in their settings, and the practical nature of frontline HCW training needs is illustrated by feedback from participants that the most applicable aspects of the training included IPC (standard and droplet precautions), recognizing COVID-19 signs and symptoms, donning and doffing PPE and effective communication and community guidance for COVID-19.

The training course met its objectives, including reaching large numbers of people in a short amount of time, and contributing to improved knowledge, skills and confidence among frontline HCW confronting COVID-19. Comparison of pre- and post-test results reflect expected geographic and demographic variation in trainee knowledge including differences in delivery modality by country and baseline clinical knowledge by cadre. In the 8 countries in which matched pre- and post-test data are available, all demonstrated statistically significant knowledge gains for all cadres and sexes at both the facility and district level.

Multiple factors including speed and flexibility contributed to successful implementation. Using a competency-based framework to guide curriculum creation allowed for ease of collaboration with MOHs and local stakeholders to adapt materials for local contexts and quickly reach thousands of individuals across the continent with the training they needed to safeguard themselves and respond to patient and community needs. Prior investments in human resources for health, including ready access to trainers and distance-learning infrastructure, facilitated the rapid roll-out of training across diverse settings. The ability to train trainers, provide tele-mentorship, and livestream trainings to remote locations improved the reach and continuity of our cascade trainings.

Several factors limit our ability to make inferences about the impact of this training. The same characteristics that enabled rapid and tailored training delivery—variation in content focus, training modality and pre/post-test design—limit the generalizability of pre- and post-test gain score results, as well as our ability to compare performance across countries and by training modality. In addition, while the post-training survey assessed participants’ responses to the training, we did not assess impact on participant behavior change or on facility-level compliance with IPC standards. Follow-up assessments which address knowledge retention, HCW skills, and facility performance would be useful to assess impact and inform future trainings; however, such assessments are outside the scope of this paper.

## Conclusion

As we learned from the 2014–2016 West Africa Ebola outbreak and as we continue to learn from present experiences with COVID-19, ongoing investment in health systems and focused attention to health workforce capacity building is critical to outbreak response. The success of our short-term IPC training initiative was due both to the speed, rigor and flexibility of its design and delivery, and to the pre-existing systems, resources, and partnerships that enabled its rapid implementation. The advent of new infectious diseases will always require new training content and curricula, but sustained impact and the ability to prepare for and respond to emerging and known disease threats requires ongoing financing and political support.

Epidemic preparedness efforts alone cannot replace the need for robust and resilient health systems including essentials such as training, management, water and sanitation infrastructure, procurement of IPC supplies, and continuous monitoring and evaluation of IPC performance. District and facility-level IPC programs offering pre-service training and in-service recertification for both clinical and non-clinical health workers, and those which include information about disease transmission, proper use of PPE and IPC protocols are also core to a safe and effective epidemic response [28].

## Data Availability

The datasets supporting the conclusions of this article are available on request from the corresponding author.

## Abbreviations

ASLM: African Society of Laboratory Medicine
CDC: Centers for Disease Control and Prevention
HCW: Healthcare workers
HRH: Human resources for health
ICAN: Infection Control Africa Network
ICAP: ICAP at Columbia University, Mailman School of Public Health
IP: Implementing partner
IPC: Infection Prevention and Control
MOHs: Ministries of Health
PPE: Personal protective equipment
RTSL: Resolve to Save Lives
WHO: World Health Organization
WASH: Water, sanitation and hygiene
