# Correction: Rapid scale-up of COVID-19 training for frontline health workers in 11 African countries

**DOI:** 10.1186/s12960-022-00765-6

**Published:** 2022-09-07

**Authors:** Fatima Tsiouris, Kieran Hartsough, Michelle Poimboeuf, Claire Raether, Mansoor Farahani, Thais Ferreira, Collins Kamanzi, Joana Maria, Majoric Nshimirimana, Job Mwanza, Amon Njenga, Doris Odera, Lyson Tenthani, Onyekachi Ukaejiofo, Debrah Vambe, Erika Fazito, Leena Patel, Christopher Lee, Susan Michaels-Strasser, Miriam Rabkin

**Affiliations:** 1 ICAP at Columbia University, New York, NY United States of America; 2 Resolve to Save Lives, An Initiative of Vital Strategies, New York, NY United States of America; 3 Mailman School of Public Health, Columbia University, New York, NY United States of America; 4 HRH Training Unit, Mailman School of Public Health, ICAP at Columbia University, 722 West 168Th Street, New York, NY 10032 United States of America

## Correction: Human Resources for Health (2022) 20:43 10.1186/s12960-022-00739-8

Following publication of the original article [1], the authors identified an error in the author name of Michelle Poimboeuf.

- The incorrect author name is: Michelle Poimbouef
- The correct author name is: Michelle Poimboeuf

The author group has been updated above and the original article [1] has been corrected.
